# Motivation for Smoking Cessation in Patients With Oral Squamous Cell Carcinoma—A One‐Time Survey

**DOI:** 10.1002/cre2.70154

**Published:** 2025-08-11

**Authors:** Lennart Johannes Gruber, Matthias Maximilian Bühler, Antonie Spillner, Stefan Andreas, Philipp Kauffmann, Henning Schliephake, Susanne Wolfer

**Affiliations:** ^1^ Department of Oral and Maxillofacial Surgery University Medical Center Goettingen Goettingen Germany; ^2^ Department of Cardiology and Pneumology University Medical Center Goettingen Goettingen Germany; ^3^ Lung Clinic and Pneumology Teaching Hospital Immenhausen University Medical Center Goettingen Immenhausen Germany; ^4^ Department of Oral and Maxillofacial Surgery Constance Hospital Konstanz Germany

**Keywords:** motivation, oral squamous cell carcinoma, smoking cessation, tobacco smoking

## Abstract

**Objectives:**

Tobacco smoking is one major risk factor in the development of oral squamous cell carcinoma (OSCC). Continuation of smoking after diagnosis and treatment is associated with an increase in recurrence rate and incidence of second tumors, with a shorter lon g‐term survival and poorer response to therapy. In the current German guideline for the treatment of OSCC, there is no clear recommendation to participate in structured smoking cessation programs.

**Materials and Methods:**

A total of 202 patients with histologically confirmed OSCC completed a one‐time assessment of their smoking behavior using three standardized questionnaires during regular tumor follow‐up. In addition to sociodemographic data, patients were asked retrospectively about their smoking habits and motivation to quit smoking before and after diagnosis and treatment.

**Results:**

A serious smoking cessation attempt before diagnosis of OSCC were stated in 54.8% of the participants. This number increased up to 82.2% after OSCC diagnosis. However, only 48.5% managed to quit smoking after diagnosis. Professional support was with only 21.92% (*n* = 16) rarely used. Motivation to quit was significantly lower before (2.75 ± 2.41) than after OSCC diagnosis (7.27 ± 2.41) (*p* = 0.001) and significantly higher among the participants who finally managed to quit (9.38 ± 1.68) than among those who continued smoking (4.79 ± 3.43) (*p* = 0.001).

**Conclusions:**

The diagnosis of OSCC appears to be an important teachable moment for smoking cessation. To maximize this effect, an early and standardized implementation of systematic smoking cessation programs into the therapeutic concept of patients with OSCC is strongly recommended.

**Abbreviations**
ETS: environmental tobacco smoke
HNSCC: head and neck squamous cell carcinoma
OSCC: oral squamous cell carcinoma
py: pack‐year
Q1: standardized questionnaire
Q2: German national healthcare guideline questionnaire for smoking cessation in patients with chronic obstructive pulmonary disease
Q3: Fagerström test for nicotine dependence
SC: smoking cessation
TS: tobacco smoking

## 1 Introduction

Among the 10 most common tumor diseases worldwide, the oral squamous cell carcinoma (OSCC) (Panarese et al. 2018) represents the most common malignant disease of the oral cavity with a share of around 90% (Chi et al. 2015). In 2020, 377.713 people worldwide developed OSCC and lip carcinoma, resulting in a total of 177.757 deaths (Sung et al. 2021).

Nicotine contained in tobacco is absorbed through the lung when smoking and through the oral or nasal mucosa when chewing or sniffing tobacco. When inhaling tobacco smoke, 25% of the nicotine reaches the brain within 7 s (Petti 2009), where it leads to a release of dopamine. This activates the reward system, which leads to the development of an addiction with psychological and physical dependence (Benowitz 2009). After a rapid increase, the nicotine concentration falls again rapidly so that addicts shortly feel the urge to smoke again (Petti 2009).

The individual risk of developing an OSCC correlates with the dose and the cumulative cigarette consumption (IARC Working Group on the Evaluation of Carcinogenic Risks to Humans, W. H. O., and International Agency for Research on Cancer 2004). The risk of former smokers is lower than of active smokers and decreases continuously with increasing duration of abstinence. In the first 4 years after quitting, the risk of cancer decreases by 35% and can even reach the risk of non‐smokers after more than 10 years (IARC Working Group on the Evaluation of Carcinogenic Risks to Humans, W. H. O., and International Agency for Research on Cancer 2004; Warnakulasuriya et al. 2010).

It should be emphasized that tobacco smoking (TS) not only promotes the development of OSCC but also is associated with an increased risk of recurrence (Khuri et al. 2001; Stevens et al. 1983; von Kroge et al. 2020) and a higher incidence of secondary tumors if consumption remains unchanged after diagnosis and therapy (Day et al. 1994; Hiyama et al. 1992; Khuri et al. 2001; León et al. 2009; Silverman et al. 1983; von Kroge et al. 2020). Additionally, continuing of smoking reduces the effectiveness of treatment interventions (Ark et al. 1997; Chen et al. 2019; Des Rochers et al. 1992; Harris and Hollstein 1993; Krutz et al. 2022) and lowers the patient survival rate (Andersen et al. 2022; Chen et al. 2019; Day et al. 1994; Fazel et al. 2021; Jerjes et al. 2012; Krutz et al. 2022; von Kroge et al. 2020). Another effect of smoking is a higher rate of postoperative wound healing complications after surgery (Gerude et al. 2014; Sørensen 2012; Vandersteen et al. 2013).

Environmental tobacco smoke (ETS) is also associated with the development of OSCC and is an additional risk factor even for smokers. It increases the risk as it does for non‐smokers or passive smokers who are surrounded by active smokers (Mariano et al. 2022; Prabhu et al. 2013; Wolfer et al. 2023).

Smokers who are unable to quit on their own need therapeutic support to achieve long‐term cessation.

Therefore, every patient should be offered ABC smoking cessation (SC) support to develop the motivation to quit smoking. This includes both quitting smoking and coping with relapse risk situations (S3 Guideline “Smoking and Tobacco Dependence” 2022).

Possible interventions to support SC could be motivational treatments and brief interventions, harm reduction, psychotherapeutic interventions, and medications for withdrawal and relapse prevention (S3 Guideline “Smoking and Tobacco Dependence” 2022).

The current German guidelines for the diagnosis and treatment of OSCC (2020) only contain a recommendation for SC as part of the treatment solely at expert consensus level (S3 Guideline “Diagnosis and Treatment of Oral Cavity Carcinoma” 2021). This contrasts with the guideline on early detection, diagnosis, and treatment of tobacco dependence. It explicitly offers psychosocial and pharmacological support for SC in patients with HNSCC, as this has been shown to be much more effective (S3 Guideline “Smoking and Tobacco Dependence” 2022).

The aim of this study was to collect date of patient's TS before the time of diagnosis and the changes in smoking behavior after diagnosis and treatment of OSCC. It is assumed that most patients are motivated to quit smoking at the time of tumor diagnosis. However, it is questionable whether this motivation is sufficient to initiate a sustainable attempt to quit and to achieve long‐term abstinence.

## 2 Materials and Methods

### 2.1 Study

This cross‐sectional observational study got approval from the local institutional ethics committee (UMG Ethics Committee; no. 27/4/22).

In total, 202 Patients with a history of OSCC were recruited from the regular tumor follow‐up between February 1, 2022, to August 31, 2022, using the following inclusion criteria: (1) age ≥ 18 years; (2) histologically confirmed OSCC; (3) tumor location of the oral tongue as the anterior two‐thirds of the tongue, gingiva of the upper or the lower jaw, floor of the mouth, palate or buccal mucosa; (4) no immunosuppression; (5) no other malignancy; and (6) capable of giving informed consent and not supervised. Exclusion criteria were defined as follows: (1) age < 18 years; (2) no histological confirmed OSCC; (3) oropharyngeal, nasopharyngeal, hypopharyngeal carcinomas, laryngeal carcinomas, and lip carcinomas; (4) history of immunosuppression; (5) history of other malignancy; and (6) not able to give informed consent and to be supervised.

Clinical data were collected from the regular clinical records, including the date of diagnosis, location of the OSCC, histology with TNM classification, if present, time of recurrence (census January 31, 2023). Of the 56.4% men and 43.6% women, 98.9% were > 40% and 43% were > 60 years of age. The most common marital status was married (66.8%), followed by single (14.4%). In terms of educational attainment, the most common qualification was main school education (43%). A university degree was found in 9% of the participants, whereas 1% had no degree. Details regarding age, sex, marital status, highest educational degree, and risk behavior are shown in Table 1.

**Table 1:** Frequencies of sex, age, marital status, and highest educational degree.

Category		Count (*n*)	Percentage (%)
Sex	Male	114	56.4
	Female	88	43.6
Age	0–40	2	1
	40–60	113	55.9
	60–100	87	43
Martial status	Single	29	14.4
	Married	135	66.8
	Divorced	16	7.9
	Widowed	18	8.9
	Allied	4	2
Highest educational degree	University	19	9.4
	Subject graduation	1	0.5
	High school diploma	9	4.5
	Tech. high school diploma	3	1.5
	Vocational college	23	11.4
	Secondary school	56	27.7
	Main school	87	43.1
	Special school	2	1
	None	2	1

Participants who stated that they had never smoked regularly were defined as non‐smokers, participants who smoked at the time of the survey were defined as active smokers, and participants who had stopped smoking before the survey were defined as former smokers. The tumor characteristics of the studied patient cohort, including tumor location, stage, and therapy received, are shown in Table 2.

**Table 2:** Collective tumor characteristics and therapy.

Category	Subcategory	Count (*n*)	Percentage (%)
Tumor location		202	
	Tongue	53	26.24
	Mouth floor	53	26.24
	Upper jaw mucosa	19	9.41
	Lower jaw mucosa	39	19.31
	Buccal mucosa	15	7.43
	Palate	16	7.92
	Retromandibular	2	0.99
	Cross‐regional	5	2.48
pT		199	
	pT1	92	46.23
	pT2	62	31.16
	pT3	20	10.05
	pT4	25	12.56
pN		199	
	pN0	141	70.85
	pN1	29	14.57
	pN2b	15	7.54
	pN2c	5	2.5
	pN3a	1	0.5
	pN3b	8	4.02
cM		199	
	cM0	197	98.99
	cM1	2	1.01
G		186	
	G1	34	18.28
	G2	136	73.12
	G3	16	8.6
R		193	
	R0	189	97.93
	R1	4	2.07
V		191	
	V0	189	98.95
	V1	2	1.05
L		191	
	L0	185	96.86
	L1	6	3.14
Pn		191	
	Pn0	188	98.43
	Pn1	3	1.57
Extranodal growth		197	
	Yes	16	8.12
	No	181	91.88
Therapy		202	
	Surgery alone	152	75.25
	Adjuvant radiotherapy	25	12.38
	Adjuvant radiochemotherapy	21	10.4
	Primary radiochemotherapy	4	1.98

### 2.2 Evaluation

Smoking behavior was recorded once during a tumor follow‐up visit using three questionnaires: a self‐developed standardized questionnaire (Q1), the German national healthcare guideline questionnaire for SC in patients with chronic obstructive pulmonary disease (Q2), and the Fagerström test for nicotine dependence (Q3) (Supporting Information).

**Figure 1:**
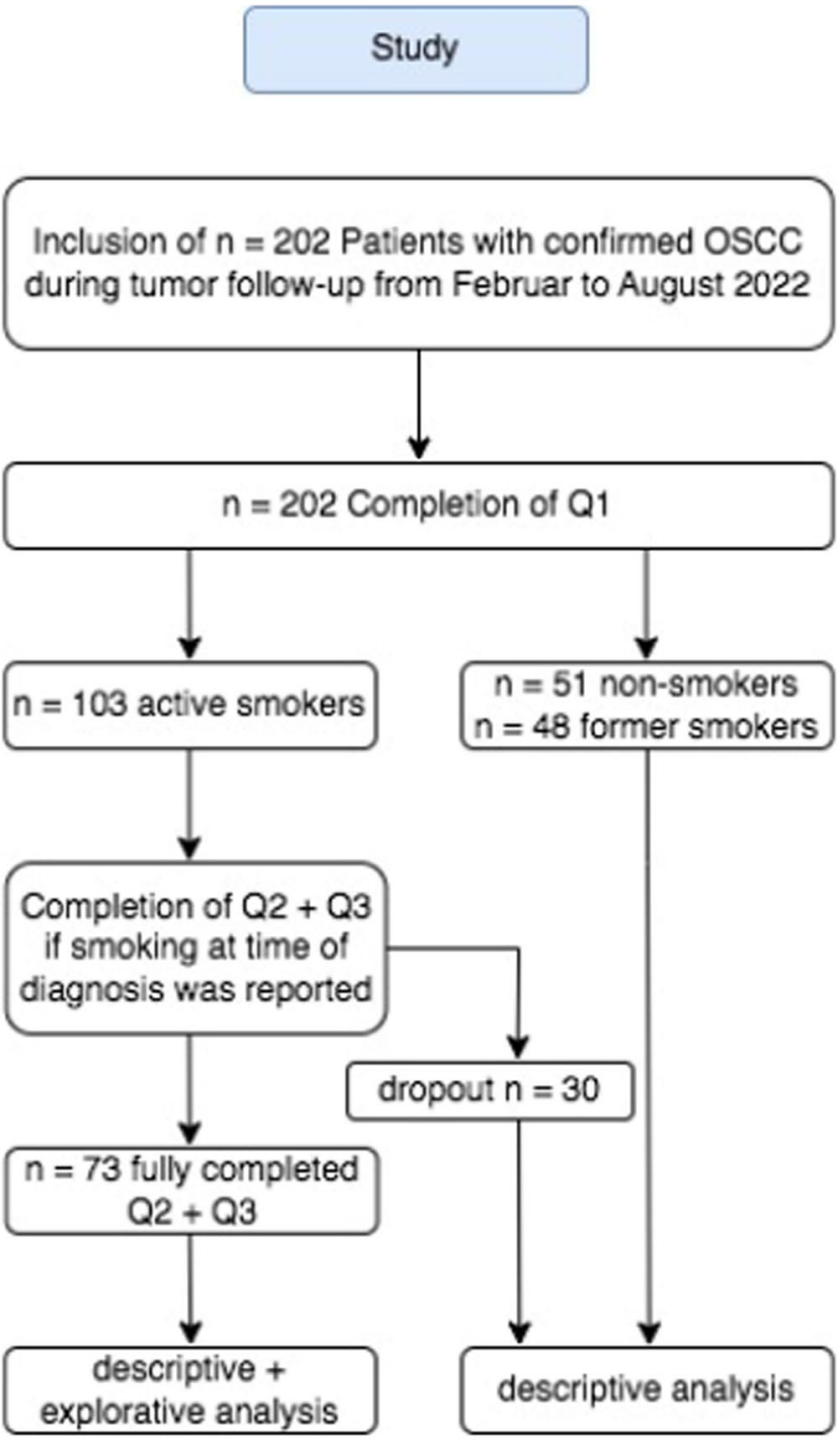
Study.

After the patient's written consent to participate in this one‐time and cross‐sectional survey, all questionnaires (Q1, Q2, and Q3) were answered by the participants in supervision of one single trained interviewer to exclude an interinterviewer variation. Starting with Q1 for all participants (*n* = 202) with collect data of former and current active and passive risk behavior, the study continued with Q2 and Q3 for participants who reported to be an active smoker at the time of diagnosis (*n* = 103). The questionnaires Q2 and Q3 were not completed by *n* = 30 patients. This left *n* = 73 for exploratory data analysis regarding SC motivation (Figure 1).

Q2 was answered to find the number of smokers in the household and at work, the previously perceived support offers for SC, the number of cessation attempts carried out so far, and the motivation for SC on a Likert scale from 1 to 10 (Andreas et al. 2023).

In our study, of the Q2 questionnaire, only the last five questions (6–10) were used because the information from the first five questions (1–5) was already collected in Q1. Questions 8 and 10 of Q2 were supplemented by a distinction between attempts to quit smoking/motivation before and after diagnosis and treatment of OSCC.

Finally, Q3 as an established score divides the degree of nicotine dependence into a score with four levels: 0–2 low, 3–4 medium, 5–6 strong, and 7–10 very strong (Heatherton et al. 1991).

### 2.3 Statistical Analysis

All patient data were collected on an individual basis and were pseudonymized before analysis. The descriptive data analysis was carried out with Microsoft Excel (Microsoft Corporation, Redmond, Washington, USA) to calculate absolute and relative frequencies or position and dispersion parameters depending on the characteristic.

The exploratory analysis examined the association between successful SC after diagnosis and treatment of OSCC and previous tobacco consumption, level of tobacco dependence, SC history, environment, demographic factors, and tumor parameters. Exploratory data analysis was performed using SPSS version 28.0 (IBM, Armonk, New York, USA). Due to the different scale levels of the variables, Mann–Whitney *U* test and Chi‐square test were performed. Normal distribution was tested using the Kolmogorov–Smirnov test. Because none of the tests showed a normal distribution, the Mann–Whitney *U* test was used exclusively for further analysis. A significance level of *α* = 0.05 was assumed for all statistical tests.

## 3 Results

**Figure 2:**
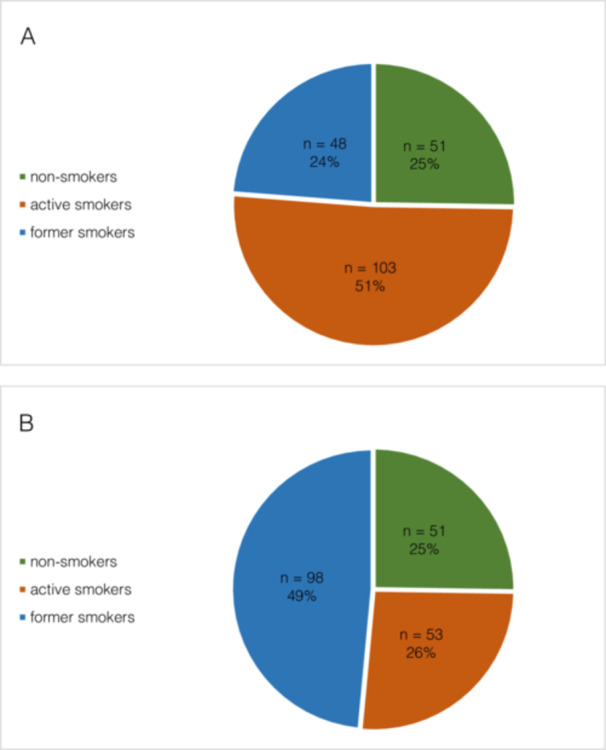
Distribution of non‐smokers, active smokers, and former smokers (A) at the time of diagnosis and (B) at the time of survey.

Patients stated that, at the time of OSCC diagnosis, 25.25% (*n* = 51) were non‐smokers, 50.99% (*n* = 103) were active smokers, and 23.76% (*n* = 48) were former smokers (Figure 2). Of the active smokers, 61.65% were mildly (*n* = 25) to moderately (*n* = 20) and 38.35% were severely (*n* = 19) to very severely (*n* = 9) nicotine dependent at the time of diagnosis. By the time of diagnosis, participants had smoked an average of 35.98 ± 12.93 years (minimum: 7, maximum: 66 years), with an average intensity of 25.87 ± 26.68 pack‐years (minimum: 0.7, maximum: 89.25).

At the time of questionnaire collection, 25.25% (*n* = 51) of all included participants were non‐smokers, 26.24% (*n* = 53) were active smokers, and 48.51% (*n* = 98) reported being former smokers, meaning that 74.75% (*n* = 151) of the participants had smoked tobacco at some time.

**Figure 3:**
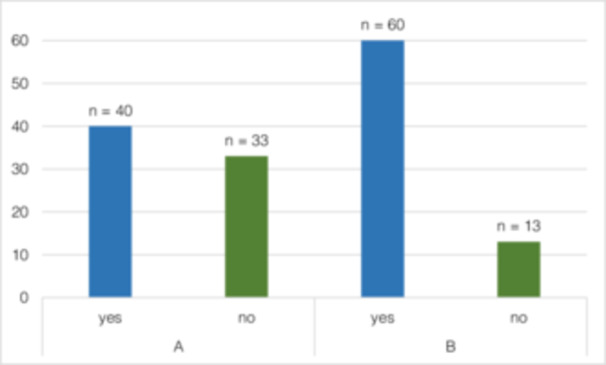
(Q2) SC attempts (A) before and (B) after diagnosis of OSCC. Significant difference between A and B (*p* < 0.001).

**Figure 4:**
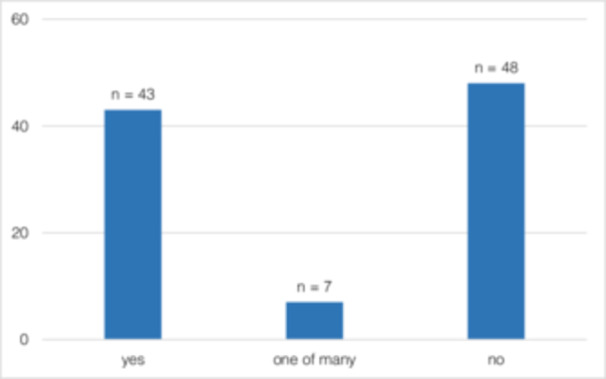
(Q1) Was the diagnosis of OSCC the reason for SC?

**Figure 5:**
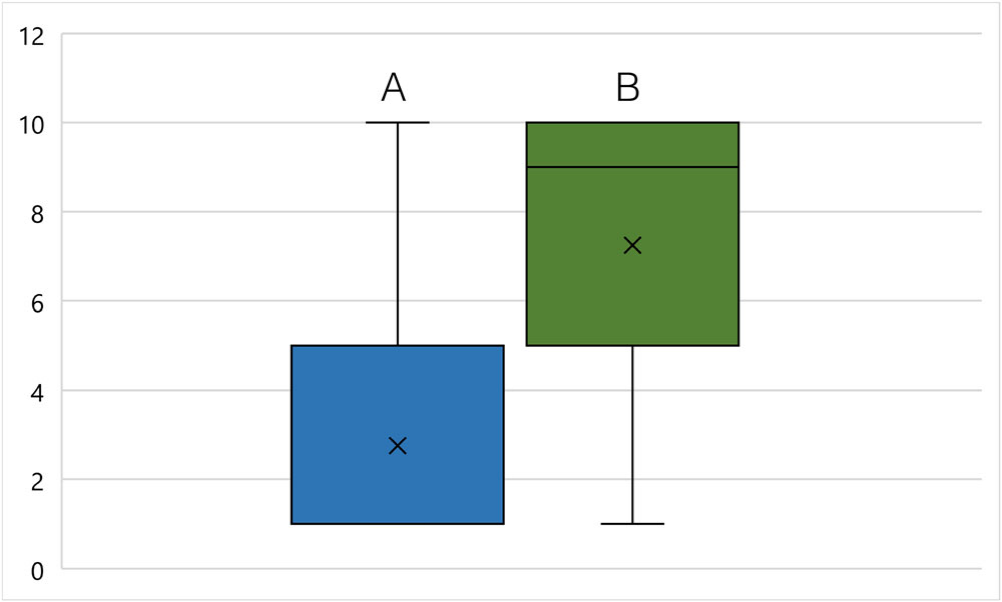
(Q2) Motivation for SC (A) before and (B) after OSCC diagnosis. 1 = lowest, 10 = highest motivation for SC. Mean (cross) for A = 2.75 and B = 7.27, standard deviation for A ± 2.41 and B = 2.41, median (horizontal line) for A = 1 and B = 9, maximum or minimum (T‐whisker), interquartile range between 25. and 75. percentile (box). Significant difference between A and B (*p* < 0.001).

**Figure 6:**
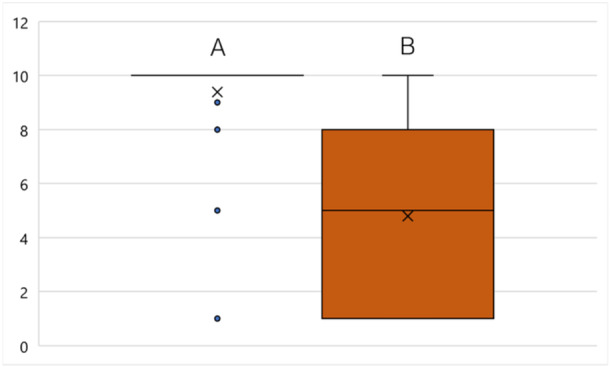
(Q2) Motivation (1 = lowest, 10 = highest) for SC at the time of diagnosis in patients with (A) successful (*n* = 39) and (B) unsuccessful SC (*n* = 43) at the time of the survey. A: Mean (cross): 9.38 ± 1.68; B: Mean (cross): 4.79 ± 3.43. Median (horizontal line) for A = 10 and B = 5, maximum or minimum (T‐whisker), interquartile range between 25. and 75. percentile (box). Significant difference between A and B (*p* < 0.001) with a strong effect size (*r* = 0.703) on quitting after OSCC diagnosis.

Although 54.79% (*n* = 40) of the participants had made an SC attempt at least once before OSCC diagnosis, after OSCC diagnosis, 82.19% (*n* = 60) (*p* < 0.001) attempted to quit smoking (Figure 3). Forty‐three patients (41.7%) stated OSCC diagnosis as the reason for SC (Figure 4). In total, 48.54% (*n* = 50) managed to quit smoking. The motivation to quit smoking was significantly lower before (2.75 ± 2.4) than after (7.25 ± 3.49) OSCC diagnosis (*p* = 0.001) (Figure 5). It was also shown that the motivation to quit smoking was significantly higher among the participants who managed to quit (9.38 ± 1.68) than among the participants who unfortunately did not manage to quit smoking (4.79 ± 3.43) (*p* = 0.001) (Figure 6). On average, 3.71 ± 5.08 SC attempts were made. Only 21.92% (*n* = 16) of *n* = 73 participants took advantage of any form of SC support.

**Figure 7:**
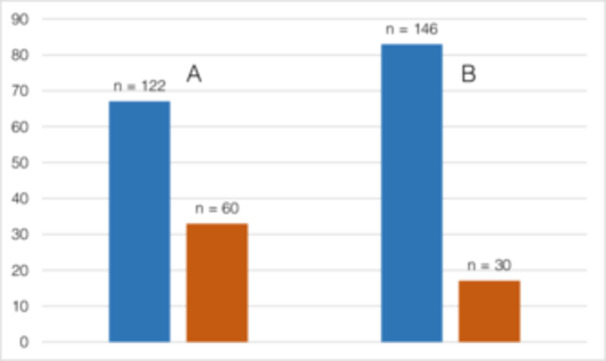
(Q1) Smoking status of patients' partners (A) before OSCC diagnosis and smoking status of patients' partners (B) at the time of the survey. Non‐smoking partners are marked in blue and smoking partners in orange with a significant difference between A and B (*p* < 0.001).

The smoking status of participants' partners before being diagnosed with OSCC and at the time of the survey differed significantly (*p* < 0.001). Although 32.97% (*n* = 60) of the partners of 182 participants were active smokers before diagnosis, after diagnosis only 17.05% (*n* = 30) of the partners of 176 participants continued smoking (Figure 7).

Demographic factors such as age and gender, smoking history, the number of attempts to quit, and the degree of nicotine dependence of the respective participants had no influence (*p* > 0.05) on successful SC. Similarly, neither the smoking behavior of the social environment nor the diagnosed tumor stage was shown to have a significant influence on smoking behavior.

**Figure 8:**
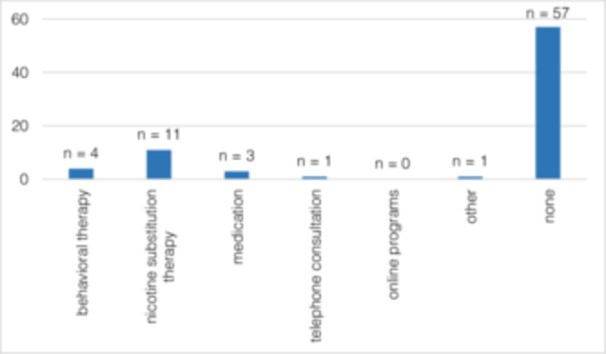
(Q2) Forms of SC support used. Multiple answering was possible. From a total of *n* = 73 participants, *n* = 77 answers were given.

Half of the patients successfully stopped smoking after diagnosis and therapy. However, professional support within the framework of SC programs was rarely used (Figure 8).

## 4 Discussion

The squamous epithelium of the oral cavity is one of the body's first points of contact with harmful substances such as tobacco smoke. Furthermore, there are multiple other effects at the oral cavity that can amplify a carcinogenic effect, including alcohol consumption, poor denture fit, and deprived oral hygiene (McDowell 2006; Ram et al. 2011). Only few studies have explicitly focused on the motivation for SC in a pure OSCC cohort (Andersen et al. 2022; Poveda‐Roda et al. 2010). Sharp et al. have already shown that a tumor diagnosis can be a teachable moment for patients because of an acute negative tobacco‐related event (Sharp et al. 2008). Conlon et al. have reported that 85.8% of 183 smoking individuals with HNSCC, wanted to quit smoking after diagnosis, which is comparable with our results (Conlon et al. 2020). This moment of motivation is critical and appears to vanish with patients starting to smoke again the longer the tumor diagnosis lies in the past (Sharp et al. 2008). According to our evaluation, SC was most frequently successful in patients with initially high motivation. Therefore, the time of OSCC diagnosis could be shown to be a teachable moment for initiating SC. According to our results, OSCC may also act as a trigger for SC according to the 3‐T‐model (tension, trigger, and treatment) as shown by Young et al. (2010).

A total of *n* = 57 (55.3%) respondents in the present study stated that they had not used any kind of professional SC support. In their review, Klemp et al. were able to show that additional SC consultation led to a significantly higher rate of SC than the usual cancer treatment alone. Furthermore, the dilemma of the definition of a valid abstinence period has been pointed out (Klemp et al. 2016).

Park et al. demonstrated in patients with tumor disease that smokers with serious SC attempts predominantly relapsed within the first 12 months after diagnosis (Park et al. 2020). With an average abstinence duration of 28.34 ± 35.54 months (median = 14 months) among participants in the present survey, it can be assumed that participants who quitted smoking after diagnosis and treatment had a good chance to be abstinent in the long term. A slight bias in the abstinence rate was expected because four participants who quit smoking after diagnosis and treatment were abstinent for less than 6 months at the time of the survey, which is the minimum time period according to the Russel Standard (West et al. 2005).

Nicotine abstinence can be assessed by self‐report or using measurement of breath CO2 levels and measurement of blood nicotine levels. Warren et al. demonstrated that abstinence rates were significantly higher in patients with HNSCC assessed by self‐report alone compared with additional biochemical validation (Warren et al. 2012). However, the problem of a sufficient definition for successful and long‐term SC continues to exist and was determined in our study purely by the patient's statement whether they were currently smoking or not. The Russell Standard, a widely used set of criteria for defining abstinence from smoking, uses the aforementioned period of 6 months to minimize the risk of bias and falsely high abstinence rates (West et al. 2005).

When measuring CO_2_ and nicotine blood levels, environmental influences cannot be ruled out. In a study with multiple regular measurements where comparative values are available for interpretation, these measurements are more valuable. Therefore, this method was not chosen in the present one‐time cross‐sectional survey.

Motivation for SC was assessed on a Likert scale of 1–10 (1 = lowest; 10 = highest). Chan et al. and van Heest et al. showed a positive effect on change of smoking behavior after diagnosis and treatment due to a high number SC attempts before diagnosis in patients with HNSCC (Chan et al. 2004; Van Heest et al. 2022). In the present study at least one SC attempt was reported by 54.8% before and 82.2% after OSCC diagnosis (Figure 3). The number of SC attempts was not found to have a significant effect on SC success.

Only one study by Poveda‐Roda et al. also focuses on patients with OSCC. This study reported a post‐therapeutic abstinence rate of 57.15%. The median duration of abstinence was 54 months (Poveda‐Roda et al. 2010). The results of the present study are of similar magnitude with a decrease in active smokers of 48.54%.

Including studies with HNSCC in the literature review, 10 additional studies can be used to compare TS abstinence after tumor diagnose with abstinence rates from 53% to 88% (Almeida et al. 2014; Chan et al. 2004; Chen et al. 2019; Duffy et al. 2008; Krutz et al. 2022; Ostroff et al. 1995; Pinto et al. 2011; Schiller et al. 2016; Silverman et al. 1983; Van Heest et al. 2022).

Compared to all other available studies of changes in tobacco consumption after diagnosis and treatment of HNSCC, the present study showed the largest number of subjects with diagnosed OSCC.

In summary, it can be stated that TS still is a hard dependency to overcome for patients with OSCC.

There are many reasons why SC attempts fail. On the one hand, depression plays an important role and is observed in up to 48% of patients with HNSCC (Schnoll et al. 2010; Simmons et al. 2013). On the other hand, sleep problems (Rieder et al. 2001; Santoso et al. 2019) and inadequate information by medical staff about the negative effects of smoking are also cited as possible reasons for failed SC attempts (Burke et al. 2009). Even after medical consultation, the most effective treatment is rarely selected (S3 Guideline “Smoking and Tobacco Dependence” 2022). Age, sex, TNM stage, dependence, setting, second carcinoma, or recurrence showed no significant effect on SC in the present study. Andersen et al. were able to show a reduced disease‐free survival with continued smoking (Andersen et al. 2022). Similarly, Jerjes et al. described that patients with successful SC after diagnosis and treatment of OSCC had a significantly reduced 3‐ and 5‐year mortality (*p* < 0.001) (Jerjes et al. 2012). In addition, severe nicotine dependence was assessed as a strong factor against patients' motivation to quit smoking and is responsible for the failure of cessation attempts (Mcbride and Ostroff 2003). In this study, the influence of the number of cigarettes smoked daily, the duration of tobacco consumption, and the number of py on successful SC could not be shown as statistically significant. Similarly, there was no correlation between dual tobacco and alcohol consumption or the degree of nicotine dependence and SC after OSCC diagnosis and treatment. In contrast, Poveda Roda et al. and Silverman et al. were able to show that patients with a large number of cigarettes smoked daily were significantly less likely to quit after diagnosis and treatment than patients with a smaller number of cigarettes smoked daily (Poveda‐Roda et al. 2010; Silverman et al. 1983).

Like the present study, Day et al. came to the conclusion that there is no significant difference in the number of py in patients with successful SC and those without cessation success (Day et al. 2020).

When additional studies of HNSCC with a smaller proportion of OSCC patients are considered, the results are inconsistent. Almeida et al. described no significant correlation between a change in tobacco use after diagnosis and treatment and the number of cigarettes smoked per day, the number of py, and the degree of nicotine dependence determined by the Fagerström test (Almeida et al. 2014). In contrast, van Heest et al. reported that unchanged tobacco use after carcinoma diagnosis and treatment was associated with a higher number of cigarettes smoked per day and longer duration of tobacco use (Van Heest et al. 2022). Furthermore, Chen et al. showed that patients with lower py were significantly more likely to quit smoking after diagnosis and treatment (Chen et al. 2019).

Regarding the influence of the patient's home and work environment, sociodemographic factors, or TNM classification, no significant correlations were found, which is largely consistent with our current data situation. However, these studies mostly investigated HNSCC collectives without a pure focus on the oral cavity with OSCC (Almeida et al. 2014; Van Heest et al. 2022). Therefore, a significant effect on SC after OSCC diagnosis and treatment of patients' home environment could not be verified. The theory that patients are more likely to achieve SC after OSCC diagnosis and treatment, if there is no peer pressure from other smokers in the household or at work, was not supported. However, HNSCC patients prefer to quit smoking if their partners and co‐workers are non‐smokers, as shown in the study by Kashigar et al. (2013). This is consistent with our findings. This study showed that smoking partners were more likely to stop smoking after the patient's diagnosis of OSCC. This raises the possibility for a teachable moment of an entire social group. Ideally, the whole patients' social environment could benefit from a systematic SC support as a holistic part of OSCC treatment.

The positive effects of SC described inevitably raise the question of how to improve patient care. The current guideline for the treatment of tobacco addiction recommends a combination of behavioral and pharmacological treatment (S3 Guideline “Smoking and Tobacco Dependence” 2022), which appears to be insufficient or underutilized in current clinical practice with smoking OSCC patients where the current guideline for the diagnosis and treatment of OSCC only recommends a consultation on SC (S3 Guideline “Diagnosis and Treatment of Oral Cavity Carcinoma” 2021). This recommendation is based on a systematic review by Klemp et al. (2016), which showed that SC counseling can increase the likelihood of abstinence by 26% and that combination with pharmacotherapy can further increase abstinence (Klemp et al. 2016). This finding is confirmed in another systematic review by Shingler et al. According to this, a combination of cognitive behavioral therapy and pharmacotherapy is associated with significantly higher abstinence rates in smokers with HNSCC (Shingler et al. 2018). These findings are further supported by a Cochrane review by Rigotti et al. that examined the effectiveness of behavioral interventions for SC in hospitalized patients with tobacco‐related diseases. Increased effectiveness was also observed when combined with nicotine replacement therapy (Rigotti et al. 2012). However, early initiation of interventions during hospitalization and at least 1 month of follow‐up after hospitalization was a prerequisite for effectiveness. Early initiation of SC therapy and follow‐up after hospitalization therefore appear to have a significant effect on SC success. In the included studies, this supportive contact was provided by telephone, letter, or e‐mail. In addition, the effectiveness of the interventions was confirmed regardless of the patient's diagnosis (Rigotti et al. 2012). Therefore, it can be assumed that these results are transferable to patients with OSCC.

In conclusion, smoking status should be routinely assessed in all patients with OSCC, and all smoking patients should receive a combination of behavioral and pharmacological treatment. Ideally, these SC interventions should be initiated as soon as possible and continued for at least 1 month after discharge from the hospital.

However, there are currently some hurdles. Kotz et al. stated that there is an urgent need for improvement in the implementation of evidence‐based interventions in SC treatment in all healthcare settings (Kotz et al. 2020). The current guideline on tobacco dependence therefore calls for the systematic implementation of evidence‐based interventions to promote SC in all healthcare facilities and to be included in the quality goals of the facilities. To address this needs, measures for counseling and treatment of smokers should be more closely integrated into the training and further education of health professionals (S3 Guideline “Smoking and Tobacco Dependence” 2022). Thus, Raupach et al. (2014) concluded that the current training of physicians regarding guideline‐compliant SC is insufficient and significantly impedes its implementation (Raupach et al. 2014). A later study of physicians in England who had received training in SC support found an effect on their own capability, perceived opportunity, and behavior (Bobak and Raupach 2018). Evidence‐based measures for SC are currently not taught as compulsory in medical studies (Raupach et al. 2013). Herold et al. were able to demonstrate long‐term effects of a training module during medical studies on knowledge, skills and attitudes toward SC measures (Herold et al. 2016). In addition, Carson et al. found that continuing education in SC measures among healthcare professionals significantly increases the likelihood of counseling and follow‐up counseling as well as long‐term abstinence rates. The training and further education of medical staff with regard to SC interventions must therefore be significantly improved to be able to offer patients adequate cessation therapies at all (Carson et al. 2012).

Due to the small proportion of patients with OSCC who are currently being introduced into systematic SC therapy, it must also be stated that the strong recommendation for a combination therapy of behavioral and pharmacotherapy in patients with HNSCC in the current guideline for the diagnosis and therapy of tobacco dependence was not transferred (S3 Guideline “Diagnosis and Treatment of Oral Cavity Carcinoma” 2021; S3 Guideline “Smoking and Tobacco Dependence” 2022). This recommendation should therefore also be included in the guidelines for the diagnosis and treatment of OSCC and replace the current recommendation (S3 Guideline “Diagnosis and Treatment of Oral Cavity Carcinoma” 2021). This could accelerate the process of a standardized implementation of SC programs in the therapy concept for OSCC and promote an improvement in the education and training of medical staff regarding SC measures.

## 5 Strengths and Limitations

This single‐center study, investigating smoking habits and motivation for SC in patients with OSCC, was subject to the limitations of a cross‐sectional survey. One limitation was the evaluation of purely anamnestic patient data. These were collected at a patient‐specific, arbitrary point in time during tumor follow‐up. The findings of this single‐center sample cannot be generalized to the entire population of patients with OSCC. A prospective study design that includes CO2 measurement to monitor smoking abstinence could achieve more valid results.

A strength of the study lay in the large population of *n* = 202 with a focus exclusively on OSCC. The use of standardized questionnaires ensured good comparability with other studies.

## 6 Conclusion

The moment of OSCC diagnosis appears to be an important influencing factor in motivation for SC. This is underlined by the described positive effects of successful SC after OSCC diagnosis and therapy. To maximize the use and the success of this motivational event for long‐term SC, an early and standardized implementation of systematic SC support into the holistic therapy of patients with OSCC seems reasonable. This was particularly supported by the large proportion of patients who failed to quit smoking after diagnosis despite high motivation.

Based on the results of this study and the findings of recent literature, it may not be sufficient to simply advise patients to stop smoking, as it is currently recommended by expert consensus in the current German guideline on the diagnosis and treatment of oral cancer. Therefore, it is proposed that systematic SC therapy should be routinely supported promptly after the first contact at the time of OSCC diagnosis with at least 1‐month follow‐up and should be included in clinical practice, and the current guidelines.

## Author Contributions

Conceptualization: Susanne Wolfer and Lennart Johannes Gruber. Methodology: Susanne Wolfer and Lennart Johannes Gruber. Validation: Lennart Johannes Gruber, Susanne Wolfer, and Antonie Spillner. Formal analysis:Lennart Johannes Gruber, Susanne Wolfer, and Stefan Andreas. Investigation: Lennart Johannes Gruber, Susanne Wolfer, and Matthias Maximilian Bühler. Resources: Henning Schliephake and Susanne Wolfer. Data curation: Susanne Wolfer and Lennart Johannes Gruber. Writing – original draft preparation: Lennart Johannes Gruber. Writing – review and editing: Lennart Johannes Gruber, Matthias Maximilian Bühler, Antonie Spillner, Stefan Andreas, Philipp Kauffmann, Henning Schliephake, and Susanne Wolfer. Visualization: Lennart Johannes Gruber and Matthias Maximilian Bühler. Supervision: Susanne Wolfer. Project administration: Susanne Wolfer. All authors have read and agreed to the published version of the manuscript.

## Ethics Statement

The study is approved by the Institutional Review Board (or Ethics Committee) of University Medical Center Goettingen (no. 27/4/22).

## Consent

Informed consent was obtained from all subjects involved in the study.

## Conflicts of Interest

The authors declare no conflicts of interest.

## Supporting information

Supplementary Submission.

## Data Availability

The data that support the findings of this study are available from the corresponding author upon reasonable request.
