# Supplementary material for: Motivation for Smoking Cessation in Patients With Oral Squamous Cell Carcinoma—A One‐Time Survey
Source: Clin Exp Dent Res. 2025 Aug 11;11(4):e70154. doi: 10.1002/cre2.70154 (PMC12337751; doi:10.1002/cre2.70154)

**Supplementary**

**Q1:** Self-developed standardized UMG-questionnaire; page 1

**Q1:** Self-developed standardized UMG-questionnaire; page 2

**Q2:** German national health care guideline questionnaire for SC in patients with chronic obstructive pulmonary disease

**Q3:** Fagerström Test for Nicotine Dependence (Q3)


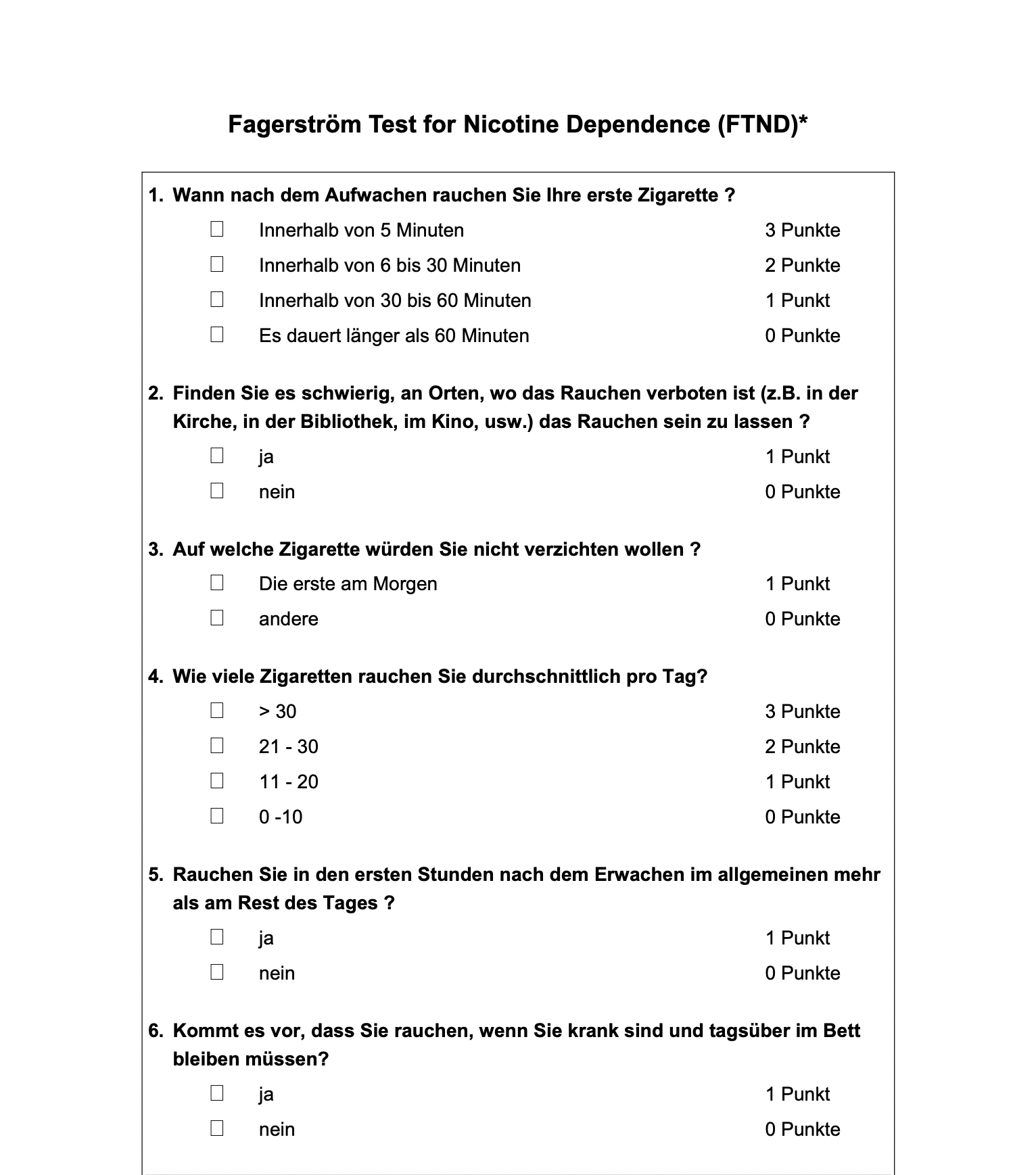

Supplement: Supplementary file 1 — Supplementary Submission. [file CRE2-11-e70154-s001.docx]
